# THE CONTRIBUTION OF FEMALE MEIOTIC DRIVE TO THE EVOLUTION OF NEO-SEX CHROMOSOMES

**DOI:** 10.1111/j.1558-5646.2012.01681.x

**Published:** 2012-10

**Authors:** Kohta Yoshida, Jun Kitano

**Affiliations:** 1 Ecological Genetics Laboratory, Center for Frontier Research, National Institute of GeneticsYata 1111, Mishima, Shizuoka 411–8540, Japan; 2 PRESTO, Japan Science and Technology Agency, Honcho KawaguchiSaitama 332-0012, Japan

**Keywords:** Centromere, female meiotic drive, multiple sex chromosome, speciation, X_1_X_2_Y, XY_1_Y_2_

## Abstract

Sex chromosomes undergo rapid turnover in certain taxonomic groups. One of the mechanisms of sex chromosome turnover involves fusions between sex chromosomes and autosomes. Sexual antagonism, heterozygote advantage, and genetic drift have been proposed as the drivers for the fixation of this evolutionary event. However, all empirical patterns of the prevalence of multiple sex chromosome systems across different taxa cannot be simply explained by these three mechanisms. In this study, we propose that female meiotic drive may contribute to the evolution of neo-sex chromosomes. The results of this study showed that in mammals, the XY_1_Y_2_ sex chromosome system is more prevalent in species with karyotypes of more biarmed chromosomes, whereas the X_1_X_2_Y sex chromosome system is more prevalent in species with predominantly acrocentric chromosomes. In species where biarmed chromosomes are favored by female meiotic drive, X-autosome fusions (XY_1_Y_2_ sex chromosome system) will be also favored by female meiotic drive. In contrast, in species with more acrocentric chromosomes, Y-autosome fusions (X_1_X_2_Y sex chromosome system) will be favored just because of the biased mutation rate toward chromosomal fusions. Further consideration should be given to female meiotic drive as a mechanism in the fixation of neo-sex chromosomes.

Sex chromosomes can undergo rapid turnover, that is, sex-linked chromosomes differ between closely related species or populations (White 1973; Charlesworth and Mank 2010). Sex chromosome turnover can occur via several mechanisms, such as the transposition of an existing sex-determination gene to an autosome (Woram et al. 2003), de novo evolution of a sex-determination gene on an autosome (Kondo et al. 2006; Tanaka et al. 2007; van Doorn and Kirkpatrick 2007; Hediger et al. 2010), and fusions between an autosome and a sex chromosome (White 1973; Charlesworth and Charlesworth 1980; Kitano et al. 2009; Kaiser and Bachtrog 2010; Kitano and Peichel 2012). Although sex chromosomes are considered stable in some taxonomic groups, such as mammals, genomic data indicate that such sex chromosomes have undergone chromosomal fusions with autosomes during their evolution (Wilcox et al. 1996; Charchar et al. 2003; Kohn et al. 2004; Charlesworth et al. 2005). Because turnover of sex chromosomes may play a substantial role in phenotypic divergence (Roberts et al. 2009) and reproductive isolation between incipient species (Kitano et al. 2009), elucidation of the forces driving sex chromosome turnover is essential to better understand the mechanisms of sex chromosome evolution and speciation.

A centromeric fusion between an autosome and a sex chromosome is one of the mechanisms by which neo-sex chromosomes evolve. Most vertebrate species with sex chromosomes have a simple male heteromorphic (XX female/XY male) or a simple female heteromorphic (ZW female/ZZ male) system. In species with the XY system, a centromeric fusion between an autosome and a Y chromosome creates an X_1_X_2_Y sex chromosome system (White 1973) (Fig. 1A), in which males have one neo-Y chromosome (a fused chromosome), one ancestral X chromosome (X_1_), and one neo-X chromosome (X_2_; a free copy of the autosome involved in the fusion), whereas females have two pairs of X chromosomes (two X_1_ chromosomes and two X_2_ chromosomes). A fusion between an autosome and an X chromosome gives rise to an XY_1_Y_2_ sex chromosome system, in which males have one neo-X chromosome (a fused chromosome), one ancestral Y chromosome (Y_1_), and one neo-Y chromosome (Y_2_; a free copy of the autosome involved in the fusion), while females have one pair of neo-X chromosome (Fig. 1A). Although such multiple sex chromosome systems can be also derived from centromeric fissions (Fig. 1B), centromeric fusions are considered to be the main mechanisms in most cases of multiple sex chromosome systems in fishes and mammals (see references in Table 1 and Kitano and Peichel 2012): there is only one mammalian case (*Wallabia bicolor*) with an XY_1_Y_2_ sex chromosome system that was likely derived from a fission of a Y chromosome (Toder et al. 1997).

**Figure 1:**
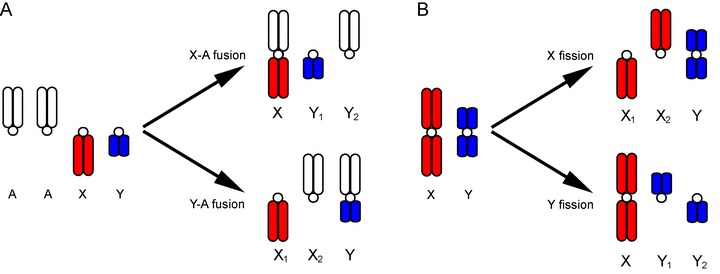
Proposed mechanisms of the evolution of multiple sex chromosome systems. Chromosome shapes during the first meiotic division are shown. (A) From a simple XY sex determination system, a fusion between an X chromosome and an autosome or between a Y chromosome and an autosome creates an XY_1_Y_2_ or an X_1_X_2_ Y sex chromosome system, respectively. (B) From a simple XY sex determination system, a centromeric fission of an X or a Y chromosome creates an X_1_X_2_Y or an XY_1_Y_2_ sex chromosome system, respectively. Autosomes, X, and Y chromosomes are indicated by white, red, and blue colors, respectively.

**Table 1:** Mammalian species with X_1_X_2_Y and XY_1_Y_2_ sex chromosome systems.

Order	Family	Genus	Species	Female 2*n*	Male 2*n*	Female NF	Male NF	Female % acrocentric	Male % acrocentric	System	References
Primates	Aotidae	*Aotus*	*azarae*	50	49	74	76	0.52	0.45	X_1_X_2_Y	(Pieczarka and Nagamachi 1988; Mudry et al. 1990)
Primates	Aotidae	*Aotus*	*boliviensis*	50	49	64	63	0.72	0.71	X_1_X_2_Y	(Ma et al. 1976; Pieczarka and Nagamachi 1988)
Primates	Aotidae	*Aotus*	*inflatus*	50	49	66	65	0.68	0.67	X_1_X_2_Y	(Pieczarka and Nagamachi 1988)
Primates	Aotidae	*Aotus*	*sp.*	50	49	74	73	0.52	0.51	X_1_X_2_Y	(Mudry de Pargament et al. 1984)
Primates	Atelidae	*Alouatta*	*belzebul*	50	49	70	70	0.60	0.57	X_1_X_2_Y	(Armada et al. 1987)
Primates	Atelidae	*Alouatta*	*fusca*	50	49	68	66	0.64	0.65	X_1_X_2_Y	(de Oliveira et al. 1998, 2002)
Primates	Atelidae	*Alouatta*	*palliata*	54	53	78	76	0.56	0.57	X_1_X_2_Y	(Ma et al. 1975)
Primates	Callitrichidae	*Callimico*	*goeldii*	48	47	72	71	0.50	0.49	X_1_X_2_Y	(Margulis et al. 1995)
Primates	Pitheciidae	*Cacajao*	*calvus*	46	45	62	62	0.65	0.62	X_1_X_2_Y	(Finotelo et al. 2010)
Rodentia	Cricetidae	*Deltamys*	*kempi*	38	37	38	38	1.00	0.97	X_1_X_2_Y	(Sbalqueiro et al. 1984; Montes et al. 2008)
Rodentia	Muridae	*Mus*	*minutoides*	36	35	36	36	1.00	0.97	X_1_X_2_Y	(Matthey 1965; Fredga 1970)
Rodentia	Muridae	*Vandeleuria*	*oleracea*	29	29	41	41	0.59	0.59	X_1_X_2_Y	(Sharma and Raman 1972)
Chiroptera	Phyllostomidae	*Choeroniscus*	*godmani*	20	19	28	26	0.60	0.63	X_1_X_2_Y	(Hsu et al. 1968; Patton and Gardner 1971)
Chiroptera	Phyllostomidae	*Mesophylla*	*macconnelli*	22	21	22	21	1.00	1.00	X_1_X_2_Y	(Baker and Hsu 1970)
Artiodactyla	Bovidae	*Tragelaphus*	*strepsiceros*	32	31	58	58	0.19	0.13	X_1_X_2_Y	(Wallace and Fairall 1968)
Carnivora	Herpestidae	*Herpestes*	*auropunctatus*	36	35	56	54	0.44	0.46	X_1_X_2_Y	(Fredga 1965a,b)
Pilosa	Megalonychidae	*Choloepus*	*hoffmanni*	49	49	58	58	0.82	0.82	X_1_X_2_Y	(Corin-Frederic 1969)
Diprotodontia	Macropodidae	*Lagorchestes*	*conspicillatus*	16	15	16	15	1.00	1.00	X_1_X_2_Y	(Martin and Hayman 1966)
Rodentia	Muridae	*Mus*	*musculoides*	18	19	36	35	0.00	0.16	XY_1_Y_2_	(Veyrunes et al. 2004)
Rodentia	Muridae	*Taterillus*	*arenarius*	30	31	36	36	0.80	0.84	XY_1_Y_2_	(Dobigny et al. 2003, 2005)
Rodentia	Muridae	*Taterillus*	*petteri*	18	19	28	28	0.44	0.53	XY_1_Y_2_	(Dobigny et al. 2003, 2005)
Rodentia	Muridae	*Taterillus*	*pygargus*	22	23	38	40	0.27	0.26	XY_1_Y_2_	(Dobigny et al. 2002)
Rodentia	Muridae	*Taterillus*	*sp.1*	22	23	40	40	0.18	0.26	XY_1_Y_2_	(Dobigny et al. 2002)
Rodentia	Muridae	*Taterillus*	*sp.2*	24	25	44	44	0.17	0.24	XY_1_Y_2_	(Dobigny et al. 2002)
Rodentia	Muridae	*Taterillus*	*tranieri*	14	15	24	23	0.29	0.47	XY_1_Y_2_	(Dobigny et al. 2004)
Chiroptera	Phyllostomidae	*Artibeus*	*jamaicensis*	30	31	50	51	0.33	0.35	XY_1_Y_2_	(Yonenaga et al. 1969)
Chiroptera	Phyllostomidae	*Artibeus*	*lituratus*	30	31	50	51	0.33	0.35	XY_1_Y_2_	(Yonenaga et al. 1969)
Chiroptera	Phyllostomidae	*Artibeus*	*toltecus*	30	31	50	51	0.33	0.35	XY_1_Y_2_	(Yonenaga et al. 1969)
Chiroptera	Phyllostomidae	*Carollia*	*subrufa*	20	21	32	33	0.40	0.43	XY_1_Y_2_	(Yonenaga et al. 1969)
Chiroptera	Phyllostomidae	*Carolia*	*perspicillata*	20	21	32	33	0.40	0.43	XY_1_Y_2_	(Yonenaga et al. 1969)
Artiodactyla	Cervidae	*Muntiacus*	*muntjak*	6	7	10	11	0.33	0.43	XY_1_Y_2_	(Wurster and Benirschke 1970)
Artiodactyla	Bovidae	*Gazella*	*granti*	30	31	60	60	0.06	0.06	XY_1_Y_2_	(Effron et al. 1976)
Artiodactyla	Bovidae	*Gazella*	*gazella*	34	35	60	60	0.29	0.29	XY_1_Y_2_	(Effron et al. 1976)
Artiodactyla	Bovidae	*Gazella*	*dorcas*	30	31	60	60	0.06	0.06	XY_1_Y_2_	(Effron et al. 1976)
Artiodactyla	Bovidae	*Gazella*	*spekei*	32	33	60	60	0.18	0.18	XY_1_Y_2_	(Effron et al. 1976)
Artiodactyla	Bovidae	*Gazella*	*leptoceros*	32	33	60	60	0.18	0.18	XY_1_Y_2_	(Effron et al. 1976)
Artiodactyla	Bovidae	*Gazella*	*subgutturosa*	30	31	60	60	0.06	0.06	XY_1_Y_2_	(Effron et al. 1976)
Soricomorpha	Soricidae	*Sorex*	*araneus Race A*	22	23	42	42	0.09	0.17	XY_1_Y_2_	(Ott 1968)
Soricomorpha	Soricidae	*Sorex*	*gemelleus*	22	23	44	44	0.00	0.09	XY_1_Y_2_	(Ott 1968)
Diprotodontia	Potoroidae	*Potorous*	*tridactylus*	12	13	22	22	0.17	0.31	XY_1_Y_2_	(Sharman and Barber 1952; Shaw and Krooth 1964)
Diprotodontia	Macropodidae	*Wallabia*	*bicolor*	10	11	18	20	0.20	0.18	XY_1_Y_2_	(Metcalfe et al. 1998)

NF = fundamental number.

Three mechanisms to promote the fixation of fusions between autosomes and sex chromosomes have been proposed. First, the presence of sexually antagonistic genes (i.e., genes with alleles that have differential fitness effects in males and females) on an autosome may drive the fusion of that autosome to an existing sex chromosome (Charlesworth and Charlesworth 1980). If a sexually antagonistic allele is present on an autosome, this allele will not easily spread within a population because of opposing selection pressures on that one allele between two sexes (Rice 1984). A translocation of the sexually antagonistic allele to a sex chromosome, either X or Y, can resolve the intralocus sexual conflict because males and females can have different alleles frequencies at the sexually antagonistic locus (Rice 1984). Second, in an inbreeding population, the presence of an autosomal locus with heterozygote advantage may promote a fusion of that autosome to sex chromosomes (Charlesworth and Wall 1999). When autosomal loci with heterozygote advantage are moved onto an X chromosome or a Y chromosome, the resulting male progeny can be heterozygous at that locus and would be favored. Third, genetic drift may promote fixation of fused chromosomes in small isolated populations (Lande 1979, 1985). Centromeric fusions often exhibit a heterozygote disadvantage (Lande 1979; King 1993) and are selected against when they are in the minority, but they would be rather favored when they are in the majority. Once genetic drift brings fused chromosomes into the majority, the derived fusions become a majority, so the fusions become more likely fixed than the ancestral karyotype. This mechanism, however, requires a very small population size (Lande 1979). In the former two mechanisms, theoretical models predict that Y-autosome fusions (X_1_X_2_Y sex chromosome systems) will be more common than X-autosome fusions (XY_1_Y_2_ sex chromosome systems) (Charlesworth and Charlesworth 1980; Charlesworth and Wall 1999). Our previous studies in fish demonstrated that X_1_X_2_Y sex chromosome systems (35/38) are more common than XY_1_Y_2_ sex chromosome systems (3/38) (Kitano and Peichel 2012). However, XY_1_Y_2_ and X_1_X_2_Y sex chromosome systems are equally common in mammals (White 1973). Thus, additional mechanisms might also have contributed to the fixation of multiple sex chromosome systems in mammals.

Here, we propose that female meiotic drive serves as another mechanism for the fixation of sex chromosome–autosome fusions. During female meiosis, only one of the four meiotic products develops into an egg. Therefore, any bias in the segregation of homologous chromosomes between the eggs and the polar bodies during the first meiotic division can have a substantial influence on the genetic composition of the progeny (Fig. 2A) (Pardo-Manuel de Villena and Sapienza 2001b; Burt and Trivers 2006; Fishman and Saunders 2008). For example, female meiotic drive may play a substantial role in mammalian karyotype evolution (Pardo-Manuel de Villena and Sapienza 2001a). Pardo-Manuel de Villena and Sapienza (2001a) compiled the karyotypes of 1170 mammalian species across disparate taxa and found that the frequencies of acrocentric chromosomes exhibit bimodal distribution patterns: species with almost exclusively biarmed chromosomes and species with almost exclusively acrocentric chromosomes are more common than expected by chance (Fig. 3A). They hypothesized that this pattern can be explained by the centromere drive (Pardo-Manuel de Villena and Sapienza 2001a). In this hypothesis, different number of centromeres in homologous chromosomes can cause nonrandom segregation (Pardo-Manuel de Villena and Sapienza 2001a) (Fig. 2B). In heterozygous carriers of centromeric fusions, transmission frequencies of the fused chromosomes (i.e., biarmed chromosomes) into eggs or polar bodies during the first female meiosis can substantially diverge from the 1:1 ratio (Pardo-Manuel de Villena and Sapienza 2001a,b). In some species, such as the mouse (*Mus musculus*), eggs have preference for chromosomes with more centromeres than polar bodies, so acrocentric chromosomes with two centromeres are more likely to be transmitted to the eggs than to polar bodies (Fig. 2B) (Pardo-Manuel de Villena and Sapienza 2001a). In other species, such as humans, polar bodies have preference for chromosomes with more centromeres than eggs, and thus fused biarmed chromosomes, which have fewer centromeres than the two acrocentric chromosomes, are more likely to be transmitted to the eggs than to polar bodies (Fig. 2B) (Pardo-Manuel de Villena and Sapienza 2001a,b). Reflecting the differences in the shape of chromosomes preferentially transmitted into eggs, all chromosomes are acrocentric in the mouse, whereas most chromosomes are biarmed in the human (Pardo-Manuel de Villena and Sapienza 2001a). Furthermore, they proposed that this bias may be caused by difference in the efficiency of centromere capture between the meiotic spindles from eggs and polar bodies (Fig. 2B) (Pardo-Manuel de Villena and Sapienza 2001b).

**Figure 2:**
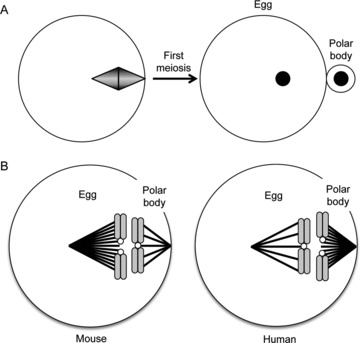
Female meiotic drive. (A) During the first meiotic division of female, only one pair of chromosomes is transmitted to the egg, whereas another pair is transmitted to the polar body. (B) A proposed mechanism of centromere-associated female meiotic drive. When the eggs have stronger spindles than the polar bodies (e.g., mice), chromosome pairs with more centromeres are more likely to be transmitted to the eggs (left panel). When the polar bodies have stronger spindles than the eggs (e.g., humans), chromosome pairs with less centromeres are more likely to be transmitted to the eggs (right panel).

**Figure 3:**
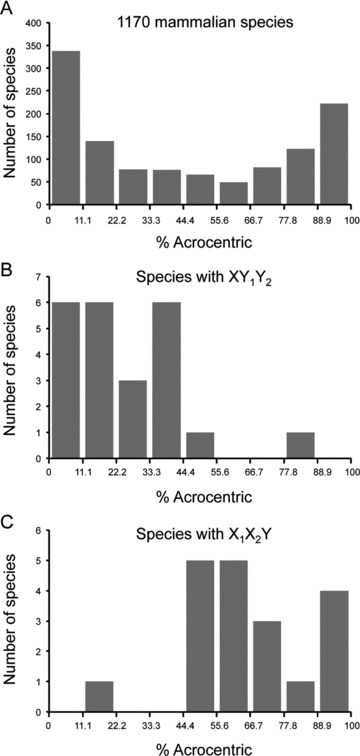
Histogram of the percentages of acrocentric chromosomes. (A) Karyotypes of 1170 mammalian species reported in Pardo-Manuel de Villena and Sapienza (2001a) are compiled. Two peaks are observed at the ends. (B) Mammalian species with XY_1_Y_2_ sex chromosome systems have more biarmed chromosomes than acrocentric chromosomes. (C) Mammalian species with X_1_X_2_Y sex chromosome systems have more acrocentric chromosomes than biarmed chromosomes.

Female meiotic drive could potentially affect the fixation of X chromosome–autosome fusions, as they would be more easily fixed in species with preferential transmission of fused biarmed chromosomes into eggs (right panel of Fig. 2B) than in species where fused chromosomes are preferentially transmitted to polar bodies (left panel of Fig. 2B). Even when X-autosome fusions occur in males, the fused X chromosomes will be transmitted to the daughters, because the sons never inherit an X chromosome from the father. This study thus hypothesizes that X-autosome fusions (XY_1_Y_2_ sex chromosome systems) occur more frequently in species with more biarmed chromosomes than in species with more acrocentric chromosomes. In contrast, Y-autosome fusions (X_1_X_2_Y sex chromosome system) occur exclusively in males and are never influenced by female meiotic drive. However, because acrocentric chromosomes, but not biarmed chromosomes, are a source of centromeric fusions, Y-autosome fusions may occur more frequently in species with acrocentric chromosomes than in species with biarmed chromosomes. Although the biased mutation rate may also favor X-autosome fusions in these species, fused chromosomes are unlikely to be transmitted to the eggs due to the female meiotic drive. Thus, while X-autosome fusions will be rarely fixed, Y-autosome fusions can accumulate in species with more acrocentric chromosomes. Thus, this study predicts that the X_1_X_2_Y sex chromosome systems would be more prevalent in species with more acrocentric chromosomes. To test these hypotheses, we compiled cases of X_1_X_2_Y and XY_1_Y_2_ sex chromosome systems in mammals and investigated the association of the two multiple sex chromosome systems with the percentages of acrocentric chromosomes.

## Materials and Methods

### DATA COLLECTION

Several cases of XY_1_Y_2_ sex chromosome systems and X_1_X_2_Y sex chromosome systems in mammals have been previously reported by White (1973). In this study, we searched the Web of Science database using several keywords, such as “XY_1_Y_2_,”“X_1_X_2_Y,”“neo-sex chromosome,”“multiple sex chromosome,”“X-autosome fusion,” and “Y-autosome fusion.” The original papers were gathered and the references therein were also checked.

For calculation of the percentages of acrocentric chromosomes, chromosome shapes were classified according to Levan's classification (Levan et al. 1964). Although there are several terms of chromosome shapes, we followed the terminology used in Pardo-Manuel de Villena and Sapienza (2001a) to be consistent with their previous work: subtelocentric and telocentric chromosomes were classified as acrocentric chromosomes, while metacentric and submetacentric chromosomes were classified as biarmed chromosomes (i.e., nonacrocentric chromosomes). The karyotype data of species with multiple sex chromosome systems were taken from the papers listed in Table 1. The frequencies of acrocentric chromosomes were calculated on the basis of female karyotype, and analysis of the male karyotype generated the same conclusions (data not shown). For comparison, the karyotype data of 1170 mammalian species were also compiled from the supplementary table of Pardo-Manuel de Villena and Sapienza (2001a).

### DATA ANALYSIS

The association between the percentages of acrocentric chromosomes and the sex chromosome systems was first tested in the species listed in Table 1 using the Mann–Whitney *U*-test. However, shared evolutionary history will likely produce correlation between karyotypes and sex chromosome systems; thus, all species are not phylogenetically independent (Felsenstein 1985). Therefore, we performed phylogenetic correction. Data were analyzed from nine phylogenetically independent pairs of species, including one species with an XY_1_Y_2_ sex chromosome system and another with an X_1_X_2_Y sex chromosome system. Tests with phylogenetically independent pairs ensure that any change in each pair reflect an independent evolutionary event (Felsenstein 1985).

**Figure 4:**
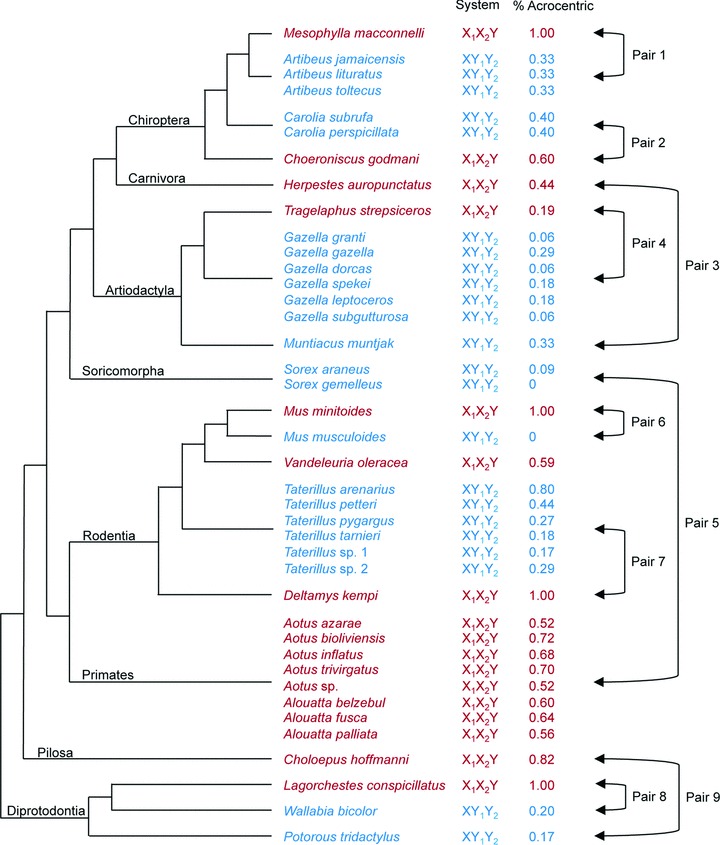
Multiple sex chromosome systems and the percentages of acrocentric chromosomes mapped on a mammalian phylogenetic tree. The XY_1_Y_2_ and X_1_X_2_Y sex chromosome systems are indicated by blue and red, respectively. The nine phylogenetically independent pairs examined in this study are indicated on the right side of the tree. The branch lengths do not reflect their divergence time.

For phylogenetic correction, a phylogenetic tree (Fig. 4) was generated using published literatures on the interorder tree (Nishihara et al. 2006), the Artiodactyla tree (Hassanin and Douzery 2003), the Rodentia tree (Jansa and Weksler 2004; Lecompte et al. 2008), and the Diprotodontia tree (Cardillo et al. 2004). The nine phylogenetically independent pairs examined are shown in Figure 4. Because the pairs 3, 5, and 9 diverge more than other pairs, these pairs may contain more events of transition than other pairs. However, the purpose of this study is not to estimate the transition rate, but to investigate the correlation between the frequency of acrocentric chromosomes and the type of multiple sex chromosome system in phylogenetically independent contrasts. Therefore, we included these three pairs for our analysis. Multiple species with the same sex chromosome systems are often monophyletic and cluster at the tip of the phylogenetic tree. For example, the genus *Gazella* consists of six species with the XY_1_Y_2_ sex chromosome system. In such cases, we performed the following two analyses. First, we calculated the average percentage of acrocentric chromosomes within that cluster. For example, in *Gazella*, the average value was determined to be 0.138. Second, only one species was randomly picked from the cluster and the association between the percentages of acrocentric chromosomes and the sex chromosome systems of the nine phylogenetically independent pairs was tested using the Wilcoxon signed-rank test. The random sampling was repeated 100 times.

## Results

We found that 23 species across five orders have an XY_1_Y_2_ sex chromosomes system and 19 species across seven orders have an X_1_X_2_Y sex chromosome system (Table 1; Fig. 4). The percentages of acrocentric chromosomes of the species with the X_1_X_2_Y sex chromosome system (Fig. 3C) were significantly larger than those of the species with the XY_1_Y_2_ sex chromosome system (Fig. 3B) (Mann–Whitney *U*-test, *U*= 25.5, *Z*=−4.88, *P* < 10^−5^). Compared to the histogram of the percentages of acrocentric chromosomes of 1170 mammalian species compiled by Pardo-Manuel de Villena and Sapienza (2001a) (Fig. 3A), the distribution of mammals with the XY_1_Y_2_ sex chromosome system was skewed to the left, while the distribution of mammals with the X_1_X_2_Y sex chromosome system was skewed to the right.

**Figure 5:**
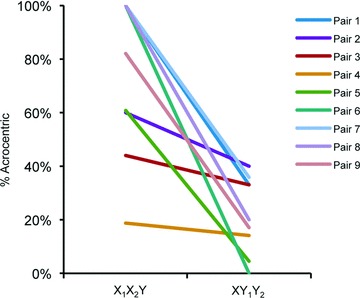
Percentages of acrocentric chromosomes of nine phylogenetically independent pairs of species with X_1_X_2_Y and XY_1_Y_2_ sex chromosome systems.

Species belonging to the same genus, such as *Artibeus*, *Carolina*, *Gazella*, *Sorex*, *Taterillus*, *Aotus*, *Alouatta*, had the same type of multiple sex chromosome system within the genus (Fig. 4), suggesting the importance of phylogenetic correction. The results of the analysis of the nine phylogenetically independent pairs did not change our conclusion. When we used the average percentages of acrocentric chromosomes for the genus with the same sex chromosome system, the percentages of acrocentric chromosomes in species with the X_1_X_2_Y sex chromosome system was significantly higher than those with the XY_1_Y_2_ sex chromosome system (Fig. 5) (Wilcoxon signed-rank test, *S*= 0, *P*= 0.0039). When we used randomly picked species from the genus with the same sex chromosome system, the percentage of acrocentric chromosomes was significantly higher in the species with the X_1_X_2_Y sex chromosome system than in the species with the XY_1_Y_2_ sex chromosome system (mean ± SE of the *P*-value of Wilcoxon signed-rank test with 100 iterations = 0.0048 ± 0.0016).

## Discussion

### DRIVING FORCES FOR THE EVOLUTION OF MULTIPLE SEX CHROMOSOME SYSTEMS

Our data support the hypothesis that female meiotic drive can contribute to the fixation of X-autosome fusions in mammals. In species with more biarmed chromosomes than acrocentric chromosomes, X chromosomes fused with autosomes may exhibit a greater likelihood of transmission to eggs than to polar bodies during female meiosis due to female meiotic drive, resulting in the prevalence of the XY_1_Y_2_ sex chromosome systems in these species. In contrast, in species with more acrocentric chromosomes, the higher number of acrocentric chromosomes could increase the probability of centromeric fusions. However, even if X-autosome fusions occur in these species, fused chromosomes are likely to be excluded from the eggs during female meiosis due to the female meiotic drive. In contrast, Y-autosome fusions occur exclusively in males and are not influenced by female meiotic drive. Thus, Y-autosome fusions can accumulate, whereas X-autosome fusions are rarely fixed in species with more acrocentric chromosomes.

The importance of female meiotic drive in mammalian X-autosome fusions does not exclude the contribution of the other three mechanisms. Rather, a combination of multiple mechanisms should influence the fixation of X-autosome fusions. For example, many cases of neo-sex chromosomes have been reported in small isolated populations of insects (Charlesworth and Wall 1999), supporting the hypothesis that genetic drift or heterozygote advantage may promote the fixation of sex chromosome–autosome fusions in these species. In addition, sexually antagonistic genes or genes controlling sexually dimorphic traits have been reported on neo-sex chromosomes in sticklebacks and cichlids (Kitano et al. 2009; Roberts et al. 2009), suggesting that sexually antagonistic selection may also play a role in turnover of sex chromosomes (Charlesworth and Charlesworth 1980; van Doorn and Kirkpatrick 2007; Veltsos et al. 2008), although we cannot exclude the possibility that the presence of sexually antagonistic alleles on neo-sex chromosomes is a consequence rather than the mechanism for the formation of neo-sex chromosomes. Consistent with the models of sexual antagonism and heterozygote advantage, X_1_X_2_Y sex chromosome systems are more common than XY_1_Y_2_ sex chromosome systems in fish (Kitano and Peichel 2012). However, in mammals, XY_1_Y_2_ sex chromosome systems are nearly as common as X_1_X_2_Y sex chromosome systems (White 1973; this study). These differences between fish and mammals may reflect taxonomic differences in the relative importance of each driving force and/or patterns of female meiotic drive. Further studies on the patterns of multiple sex chromosome systems and female meiotic drive across diverse taxa will contribute to a better understanding of the mechanisms underlying the variation in the prevalence of different multiple sex chromosome systems.

### FEMALE MEIOTIC DRIVE AS AN EVOLUTIONARY FORCE

Understanding the contribution of female meiotic drive to sex chromosome–autosome fusions is important because sex chromosome turnover may promote phenotypic divergence and the establishment of reproductive isolation between species. First, abnormal segregation of fused chromosomes during male meiosis can cause hybrid sterility (King 1993). In addition, sex chromosomes can play special roles in speciation, because sex chromosomes have several unique characteristics of transcriptional regulation (Ohno 1967; Vicoso and Charlesworth 2006), such as dosage compensation (Wu and Xu 2003; Livernois et al. 2012) and inactivation of X chromosomes during early spermatogenesis (Presgraves 2008), and the efficacy of selection (Rice 1984; Vicoso and Charlesworth 2006). Although empirical data demonstrate that established sex chromosomes may play a special role in speciation (Coyne and Orr 2004; Presgraves 2008; Qvarnstrom and Bailey 2008), little is known about the roles of neo-sex chromosomes in speciation, except in a few cases (Kitano et al. 2009). Further studies on the role of neo-sex chromosomes in phenotypic divergence and reproductive isolation across diverse taxa will lead to the elucidation of the roles of sex chromosome turnover in speciation.

Female meiotic drive can play an important role in many evolutionary processes in addition to the turnover of sex chromosomes. Female meiotic drive can also contribute to karyotype evolution of autosomes (Pardo-Manuel de Villena and Sapienza 2001a), and divergence in karyotype of autosome can contribute to reproductive isolation between species (King 1993). In species with ZW sex chromosomes, female meiotic drive can cause sex ratio bias (Rutkowska and Badyaev 2008). The fixation of supernumerary chromosomes (B chromosomes) may also be influenced by female meiotic drive (Bidau and Martí 2004; Palestis et al. 2004). Although centromere drive is one of the proposed mechanisms of female meiotic drive (Henikoff et al. 2001; Malik and Bayes 2006; Fishman and Saunders 2008), little is known regarding the molecular mechanisms of female meiotic drive. The elucidation of the molecular mechanisms of female meiotic drive is imperative for a better understanding of sex chromosome evolution and speciation.

## Conclusions

Female meiotic drive plays an important role in the evolution of X-autosome fusions in mammals. Because sex chromosome–autosome fusions can contribute to important evolutionary processes, such as speciation, further molecular studies should be conducted to elucidate the mechanisms of female meiotic drive. The relative contribution of female meiotic drive and patterns of female meiotic drive may vary between taxa. It is, thus, imperative to investigate the prevalence of different multiple sex chromosome systems and patterns of female meiotic drive across diverse taxa for a better understanding of neo-sex chromosome evolution in animals.
